# A young child with a history of wheeze

**DOI:** 10.1038/s41533-017-0020-3

**Published:** 2017-03-16

**Authors:** James Paton, Patrick Bindels, Ann McMurray, Jodie Biggins, Rebecca Nantanda, Marianne Stubbe Østergaard

**Affiliations:** 1 School of Medicine Dentistry & Nursing, Child Health, College of Medical, Veterinary, and Life Sciences, Queen Elizabeth University Hospital, University of Glasgow, Zone 1, Ground Floor, Office Block, Glasgow, UK; 2 Department of General Practice, Erasmus Medical Centre, Rotterdam, Netherlands; 3 Department of Respiratory and Sleep Medicine, Royal Hospital for Sick Children, Sciennes Road, Edinburgh, EH9 1LF Scotland UK; 4 Patient and Public Involvement Group, Asthma UK Centre for Applied Research, Usher Institute of Population Health Sciences and Informatics, University of Edinburgh, Doorway 3, Medical School, Teviot Place, Edinburgh, EH8 9AG UK; 5 Makerere University Lung Institute Makerere University College of Health Sciences, Kampala, Uganda; 6 The Research Unit for General Practice and Section of General Practice, Department of Public Health, University of Copenhagen, Copenhagen, Denmark

## Abstract

The parents of a 3-year old boy are anxious about their son who has recurring episodes of wheezing. They are frustrated that no one seems to be able to give them answers to their questions and would like a referral to a specialist. Does their son have asthma and what is the prognosis; how can the recurrent wheezing be managed and can the risk of asthma be reduced; are there lifestyle changes that could improve the environment and avoid triggers? Communication and support from the family practice team were essential. Listening to the parents’ concerns, explaining the diagnostic uncertainty, being realistic about what drug treatments could achieve, and providing practical advice on inhaler use and trigger avoidance reassured the parents that there was a strategy for managing their son’s wheeze. The specialist referral was postponed.

Case study

- The parents of a 3 year old boy have come to see their family doctor about their son whose recurrent episodes of wheeze and respiratory symptoms are causing concern. They are frustrated that no-one seems able to give them a clear diagnosis, and worried because the treatment they have been given is not controlling his symptoms. They would like a referral to a specialist to see if they can get some answers.
- Looking back through the records, the first episode of wheeze was at the age of 6 months. This was diagnosed as bronchiolitis and resulted in a brief admission. He had one further admission the following winter that was labelled as ‘viral associated wheeze’, and two subsequent attendances at the emergency department, on the last occasion being told that he had ‘asthma’. In addition there are six primary care consultations for ‘wheeze’ or ‘chest infections’. These episodes have been variously treated with bronchodilators, steroids (inhaled and oral) and/or antibiotics—none of which have had any convincing effect.

- He was a normal full term delivery and thrived well from birth. Mother had hay fever as a teenager, and father smokes (though ‘never in the house’)

A clinical case, such as this 3-year old boy with recurrent wheeze, raises many questions that need to be explored in order to address parental concerns and manage the child’s condition. The boy is thriving, height and weight just above the 50th centile.

## Why do the parents request a referral?

A host of reasons may underpin the parent’s request. Is their concern that the wheezy episodes are harmful? Are they unable to sleep because the child is disturbed at night? Is it the lack of a clear diagnosis and uncertainty about the boy’s long-term prognosis that is causing the worry? Do the parents want advice on avoiding triggers or information on what to do when the wheezing recurs, or is the main focus of this consultation the (perceived) additional value of a referral to a specialist? Or all of these?

## Does our son have asthma, and what is the prognosis?

### Wheezing is common in young children

Wheezing in children under 3 years of age is common. By 30 months, 26% of children in a UK birth cohort (ALSPAC, Avon longitudinal study of parents and children) had wheezed in the previous 12 months.^1^ Wheezing, as in this case, is often not just a minor inconvenience. Data from the latest British Thoracic Society national paediatric audit of wheezing/asthma showed that 24% of all the children admitted to hospital were between 12 and 24 months, boys outnumbering girls in a ratio of nearly 2 to 1.^2^

Longitudinal birth cohort studies have transformed our understanding of wheezing in early childhood,^3^ demonstrating that the origins of most asthma lie in early childhood and that variations in the natural history of childhood wheezing are associated with different long-term outcomes. Between 4–6 of these ‘phenotypes’ have been identified.^4, 5^ One major group is transient early wheezers whose symptoms remit by the time the child is school age. The absence of atopy is, at present, the best marker for this group.^5^ Early onset of wheezing is associated with lower lung function at adolescence and the presence of atopy is associated with persisting asthma.^4^ However, at present, it is not possible to assign a particular phenotype to an individual child to determine either treatment or prognosis; indeed, it is common for the features to change during early childhood.^5^ A summary of the recent evolution of the terminology of 'asthma' in children is given in Table 1.

**Table 1:** Asthma: What’s in a name?

Historically, there has been a reluctance to diagnose asthma in children. In 1983, Speight et al., highlighted that children who were not given a diagnosis of asthma were not treated appropriately and suffered unnecessary morbidity.^i^ Fears that the label of ‘asthma’ might cause distress were unfounded; parents were ‘uniformly relieved’ that the cause of their child’s symptoms had been identified. There followed a drive to reduce under-diagnosis and under-treatment, though the status of wheezy infants (under 1 year of age) remained contentious.^ii, =iii^ Studies of the natural history of asthma in children,^iv^ however, began to define phenotypes of ‘transient early wheezers’, ‘late-onset wheezers’ and ‘persistent wheezing’ which seemed to contradict the drive to ‘encourage healthcare professionals to make a positive diagnosis of asthma whenever recurrent wheezing, breathlessness and cough occur’,^iii^ by suggesting that only a minority of wheezy toddlers would prove to have persistent asthma. The concern now was over-diagnosis and over-treatment of young children with guidelines highlighting the ‘difficulty of making a confident diagnosis of asthma in young children’.^v^
In some healthcare contexts under-diagnosis of asthma remains a problem, as respiratory symptoms are routinely labelled (and treated) as pneumonia,^vi^ or described symptomatically to avoid the perceived stigma of the label ‘asthma’.
This case study has adopted a pragmatic approach, sharing uncertainties of diagnosis and prognosis with the parents, objectively monitoring trials of treatment so that symptoms that can be treated are relieved, a strategy that resonates with the contemporary approach of ‘treatable traits’.^vii^
i. Speight, A. N. P., Lee, D. A., & Hey, E. N. Underdiagnosis and undertreatment of asthma in childhood. *BMJ* **286**, 1253–1256 (1983).
ii. British Thoracic Society. The British guidelines on asthma management. *Thorax* **52 (**Suppl. 1), 1–21 (1997).
iii. Global Initiative for Asthma. Global strategy for asthma management and prevention. GINA (1995) Diagnosis: pp 47–61
iv. Martinez, F.D., Wright, A.L., Taussig, L.M., Holberg, C.J., Halonen, M., & W.J. Morgan. Asthma and wheezing in the first six years of life. The Group Health Medical Associates. *N. Engl. J. Med*. **332,** 133–138 (1995).
v. Global Initiative for Asthma: Global Strategy for Asthma Management and Prevention, 2016. Available from www.ginasthma.org. Accessed July 2016
vi. Nantanda, R., Tumwine, J. K., Ndeezi, G., & Ostergaard, M. S., Asthma and Pneumonia among children less than five years in Mulago hospital Uganda: evidence of under-diagnosis of asthma. *PLoS One* **8**, e81562 (2013).
vii. Agusti, A., Bel, E., Thomas, M., Vogelmeier, C., Brusselle, G., Holgate, S., *et al*. Treatable traits: toward precision medicine of chronic airway diseases. *Eur. Respir. J.* 47, 410–419 (2016).

### Defining ‘wheeze’ is not always simple

Wheezing is usually associated with airflow obstruction and is central to the diagnosis of asthma. In older children, airflow obstruction and reversibility can be documented objectively on spirometry or peak flow measurements, but lung function measurements in children younger than 4–5 years are usually not feasible in clinical practice. This places much greater emphasis on the history of wheezing provided by the parents/caregivers, which is problematic because what parents and doctors mean by ‘wheezing’ is often very different.^6, 7^ (See Table 2). Furthermore, the word ‘wheeze’ does not exist in some languages. Because of this most guidelines emphasise that if a doctor hears wheezing on auscultation, it is an important observation to record.

**Table 2:** Parents interpretation of children’s respiratory symptoms.^6^

A study in the East end of London, invited parents (first language English, Urdu, Bengali (Sylheti), or Turkish) to view a video of children’s respiratory symptoms:
• A third of parents use other words for wheeze; a third falsely label other sounds as wheeze
• Compared to other respiratory sounds, parents are more likely to label wheeze correctly
• Parents are better able to locate sounds than to label them
• There was no significant difference between parents of wheezers and non-wheezers in accuracy of labelling of location
• Parents are better at labelling if English is their first language

### All that wheezes is not asthma

Not all wheezing is due to asthma or viral infections. There are rare but important differential diagnoses.^8^ Persistent wheezing in young children, wet cough, vomiting, failure to thrive and a poor response to anti-asthma medications may be important clues to alternative diagnoses such as cystic fibrosis.

### Viral wheezing and asthma

Viruses have been found in at least 80% of wheezing episodes in children.^9^ With the exception of respiratory syncytial virus (RSV) in infants hospitalised with bronchiolitis, human rhinovirus (HRV) is by far the most common virus isolated in children over 12 months.^10^ HRV-C is the most common rhinovirus species in wheezing children, and, compared to other viruses, is more often associated with recurrent acute wheezing attacks severe enough to result in children presenting to hospital.^11^ In the follow up of a high-risk birth cohort (one parent with positive skin prick test to an aeroallergen and/or asthma) the persistence of asthma at age 13 years was most strongly associated with rhinovirus-associated wheezing illnesses and with aero-allergen sensitisation in early life.^12^ Evidence suggests that some children are more susceptible and have more severe rhinovirus infections because of a subtle defect in innate anti-viral immunity.^13^

### Bronchiolitis and the link with asthma

In the first year of life, bronchiolitis (usually defined as the first episode of wheezing in children less than 2 years old)^14^ due to RSV infection is the commonest lower respiratory tract illness with wheezing, affecting around 1 in 3 children.^15^ On auscultation there is wheezing and/or fine crackles. The disease is usually mild with only 2–3% of children being hospitalised.^14^

An association between bronchiolitis and asthma has been noted in many studies. However, while longitudinal follow-up suggests that RSV infections in early childhood are associated with an increased risk of wheezing, this association subsides with age and becomes insignificant by 13 years, unlike rhinovirus infections where the association with wheezing persists.^16^

### Will symptoms persist in the long term (the prognosis)?

For some children, early wheezing will translate into long-term asthma. This is particularly the case for those with early rhinovirus infections, with sensitisation to aeroallergens and with reduced lung function. The problem is that we cannot, with complete certainty, identify those in whom symptoms will persist and those in whom they will remit. Nevertheless, in the child in our case study, the early frequent and severe episodes in a child with a maternal history of hay fever and possible exposure to environmental smoke may point to a more protracted course of the respiratory symptoms.

Although not widely used in clinical practice, several asthma prediction scores have been developed and published in the last decade,^17^ and may usefully inform the history that the healthcare professional needs to take. The clinical asthma prediction score (CAPS), designed specifically for use in general practice, is based on five parameters: age, family history of asthma or allergy, wheezing-induced sleep disturbances, wheezing in the absence of colds, and (if available) specific Immunoglobulin E.^18^ The score ranges from 0 to 11 points; CAPS <3 signifies a negative predictive value of 78% while CAPS ≥7 signifies a positive predictive value of 74%. Measurement of specific IgE provides additional value, though the downside is the need for a blood test in a young child.

### Prognosis for the 3 year old boy in the case study

Based on the available information, the score in this boy will be at least three (asthma probability 30% at school age) but, with additional information on specific IgE and sleep disturbances, could be as high as nine (asthma probability of 82% at school age). These CAPS scores suggest a policy of either watchful waiting (asthma probability 30–60%) or initiating formal asthma management (asthma probability of 60% or higher).^18^ The wide range reflects the current difficulties in predicting prognosis.

## What treatment will reduce our son’s symptoms—and, if possible, prevent long-term asthma?

At present, there is no treatment known to ‘cure’ asthma. Current treatments, however, can control symptoms and modify the chances of attacks.^19, 20^ Bronchodilators should be used when the child is wheezy, though discussion with parents is important to ensure they are interpreting sounds correctly and that the child responds to the broncholdilator. If asthma is probable, inhaled steroids are the most effective treatment for controlling symptoms and should be first-line treatment if attacks are frequent and severe and/or if there are interval symptoms. Perhaps the one clear ‘fact’ is that complete avoidance of exposure to environmental tobacco smoke is important.^21^

### Does preventing RSV infection reduce risk of asthma?

Reflecting the observation that the persistence of wheezing beyond childhood is associated with rhinovirus infection (as opposed to RSV), prevention of RSV infection does not have a measurable effect on subsequent episodes of wheeze and asthma.^22^ Giving pre-term infants anti-RSV antibodies for the first year of life reduces RSV infections but not recurrent wheeze over the pre-school years.^23^

### Does early start of inhaled steroids prevent risk of asthma?

In 2006 three studies were published on the use of inhaled corticosteroids (ICS) in young children at high risk of developing asthma with one or more episodes of wheeze.^24–26^ Although some children had a temporary reduction in symptoms during ICS treatment, this did not prevent development of asthma. So, in our clinical case, it is important to discuss with the parents that the early start of ICS is not needed as a primary prevention strategy but it might have an effect on the severity of the symptoms.

### Relief of acute wheezy episodes

Let us consider that the consultation for this boy and his parents was triggered by a further attack of respiratory symptoms and wheeze. If there are no alarming symptoms, such as respiratory distress, requiring immediate intervention and/or referral, the use of a short acting beta_2_ agonist (SABA) will be the drug of choice to relieve the acute wheeze.^8^ Symptom relief is the main goal; SABA do not alter the natural course of the wheezy episode.

SABA can be administered safely and effectively at all pre-school ages, including below the age of one. Inhalation is well tolerated and an effect can be expected within 10–15 min. If necessary, inhalation of SABA (with a face mask) during the consultation may provide prompt relief of symptoms, demonstrating both how inhaled medication should be delivered by a spacer (five breaths to one puff) and the rapid symptom response that can result. A dose of a SABA may be needed every 3–6 hours for one or more days until the symptoms of wheeze disappear.

It is essential to ask the parents to revisit your practice at the end of the episode of respiratory symptoms (normally 1–2 weeks after the first visit). During this review the effect of the medication can be evaluated, and in case of complete remission of the symptoms medication should be stopped in order to prevent unnecessary use and overtreatment with SABA in the future. Furthermore, the parents can be advised on when to visit the practice again in case new symptoms appear.

### Prevention in children with frequent wheezy episodes or a higher probability of asthma

**Fig. 1:**
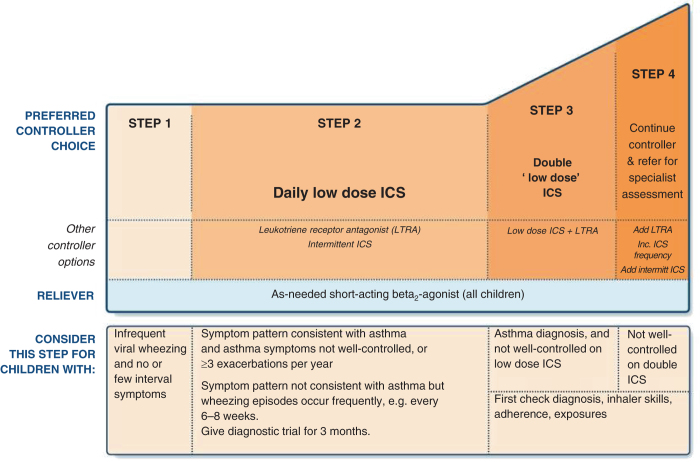
Stepwise approach to pharmacotherapy in children under 5 years. Reproduced with permission from the GINA guidelines.^8^

The indication for treatment with ICS (step 2 in GINA; see Fig. 1) is based on the frequency and severity of symptoms, and the probability that the child has asthma. The older the child, the presence of a multiple trigger wheeze and the presence of a positive specific IgE test to house dust mite, cat or dog allergens (or a positive family history for asthma and /or allergy) will increase the chances of a response to regular treatment with an ICS.

Treatment with ICS should be started as a carefully monitored diagnostic trial,^27^ and the clinical effect evaluated after 4–8 weeks.^28^ If the child responds well to a treatment with ICS, it is recommended, in discussion with parents, to reduce and ultimately withdraw the medication to exclude natural resolution of symptoms. If symptoms recur during or after withdrawal, restart treatment and consider on-going treatment.^8, 29^ If there is no response to treatment, the ICS should be stopped and alternative diagnoses should be reconsidered.

The use of ICS in children with viral induced episodes of wheeze, without symptoms or triggers in between episodes, is more controversial. The effect on symptoms is at best limited, but a recent meta-analysis has shown that short-term (7–10 days) high dose ICS, starting at the first sign of an URTI, may reduce the risk of severe exacerbations.^20^

A follow-up consultation in general practice is essential when inhalation medication is started in any child irrespective of the indication. During this review, the parents can be informed about the short and long-term prognosis and the action to take during a subsequent episode. Prevention of overtreatment of children with ICS and the (in)correct labelling of wheeze as asthma is as important as overlooking an asthma diagnosis and not treating with ICS. Until an asthma diagnosis is confirmed by a physician, requests for repeat prescriptions of SABA or ICS in preschool children should trigger a review of current status.

### Other medication that may be considered

Oral steroids during attacks do not seem to be effective in preschool children with viral induced wheezing of moderate severity.^30, 31^ They should be used only in children with severe wheeze, and even then the evidence is not robust.^19^

A recent Cochrane review revealed no benefit from the use of leukotriene receptor antagonists (LTRAs) in pre-school children with viral induced wheeze. However, identifying these phenotypes is challenging, and individual children may warrant a carefully monitored trial of a LTRA.^32^ Long acting beta_2_-agonists (LABA) have been studied in older children with persistent asthma as add-on therapy to ICS. There are no studies on the use of LABA in preschool children with recurrent wheeze and therefore their use cannot be recommended.^33^

### Management strategy in the 3 year old boy in the case study

In this particular case, the GP will need more than one review visit before a clear clinical picture will emerge and before the parents can be informed about the likely prognosis of the respiratory symptoms in their child. The parents reported that inhaled steroids did not have a convincing effect, but parental expectations need to be discussed (e.g. ICS will not reduce the frequency of viral upper respiratory tract infections), and inhaler technique and compliance with the trial of treatment needs to be checked and discussed (see below). If the child is currently symptomatic a further carefully monitored trial of ICS should be considered, if they are asymptomatic a wait-and-see strategy may be appropriated. In either situation, even after reassurance of the parents either by the General Practitioner (GP) and the practice nurse, a referral to a specialist may sometimes be appropriate as the most effective way to reassure the parents. Depending on resources in primary care, however, a paediatrician may not have more diagnostic or therapeutic possibilities in preschool children than a general practitioner.

## Supporting worried parents

### Diagnostic uncertainty & dealing with parental anxiety

It is recognised that approximately one in three children has at least one episode of wheeze before their third birthday,^1, 3^ with the expectation that the majority of children will outgrow their symptoms between age 3 and 8 years.^1, 3, 34, 35^ This however provides little comfort to a parent whose child is exhibiting symptoms and experiencing exacerbations (see a perspective from a parent in Table 3). It is important to explain that diagnosis is based on the clinical history, symptoms and response to treatment, and that these will need to be carefully observed and re-considered over time.

**Table 3:** Perspective from a parent

*Getting a diagnosis*
It started with bronchiolitis in autumn when my son was under 2 years old however he had subsequent wheezy episodes over winter and spring and the diagnosis changed to viral induced wheeze. Depending on who we see, either in accident and emergency, GP practice or hospital consultant some say he may have asthma and others say he is too young to have asthma. That has left us as parents frustrated. We have a family history of asthma so it could be that.
*Medications*
When my son has a wheezy episode we have a plan to follow which was given to us by the respiratory nurse specialists at the hospital. It gives us guidance on what to do when he gets a cold and when we need to see someone. Prednisolone seems to work but he has had so many courses over the last year some of the doctors have started to admit him and monitor him instead without giving steroids.
Giving the inhaler through the spacer with mask has been challenging. Sometimes nurses don’t do it correctly or are in a rush to give all 10 puffs. This has scared my son in the past but he got used to it over time. We know that if he is crying or upset he won’t get a full dose so it is important to keep him calm. We learned some distraction techniques from the nurse specialists and they have been helpful.
*Impact on family life*
We have been to see our general practitioner, accident and emergency department, or hospital consultant on many occasions. The unpredictability of the episodes has made it difficult for us to make family plans especially for a holiday. We ended up in hospital on two occasions when we were away from home. We have had to take time off when he is unwell as he cannot go to nursery and this has had an impact on our jobs. Medical staff keep saying it will get better as he gets older.

Treatment has to be tailored to the individual child. Parental acceptance that not all asthma therapies will prove effective in reducing exacerbations can be hard to achieve. Explanations of pharmacological treatment limitations may help to achieve more realistic expectations. Some parents may need longer consultations or will benefit from a referral to other members of the healthcare team such as the health visitor, asthma educator, respiratory nurse, physiotherapist, or community health worker. Education needs to be provided in plain language, using pictures or models to illustrate, and tailored to the parents’ current understanding and beliefs. Parents may also benefit from speaking to other parents who have been in a similar situation and they may seek this type of support via social media such as Facebook or Twitter. Parents should always be cautioned about the use of less reputable sources of self-help and encouraged to discuss strategies for self-management with their own doctor or nurse. Table 4 lists some useful websites for families.

**Table 4:** Useful websites for families of wheezy children

Organisation	Website	Description
Asthma UK	www.asthma.org.uk	Advice and support parents need to help their child stay well with their asthma
Chest Heart & Stroke Scotland.	www.mylungsmylife.org	Information, tips and advice to help parents make choices about their child’s asthma
Children and Young Peoples Allergy Network Scotland	www.cyans.org.uk	The ‘families’ section gives basic information on the different types of allergy and how to manage allergies
European Lung Foundation	http://www.europeanlung.org	Reliable information about a range of lung diseases and their risk factors

### Inhaler technique

The first experience of administering inhaled medication via a spacer can have an impact upon the child’s acceptance of future treatment. The spacer is often used for the first time when the child is experiencing difficulty breathing. Having a facemask placed over their nose and mouth can be frightening. Prior to first use the child should have an opportunity to handle the spacer and build up to the facemask being kept in position for up to 10 s dependant on taught technique. Between wheezy episodes parents should ensure their child remains familiar with the spacer to try and avoid future distress. Although actively accepting an inhaler should be the goal, administering treatment while a child is sleeping is a practical strategy that may help in some situations. Small children should never be chastised or wrapped in blankets or towels to aid with inhaler administration and these methods should be replaced with praise and distraction techniques. Holding techniques should be demonstrated and parents should be signposted to websites with demonstration videos as reminders (for example: Asthma UK ‘Using your inhalers’ https://www.asthma.org.uk/advice/inhalers-medicines-treatments/using-inhalers). Inhalers (and spacers) should only be prescribed after patients have received training in the use of the device and have demonstrated satisfactory technique.^27^ If this is difficult in a time-limited consultation, arrangements may be made with a local community pharmacist, healthcare assistant, health educator or practice nurse to check inhaler technique when inhalers have been prescribed.

### Self management

There is a paucity of evidence about effective self-management strategies for parents of pre-school children.^27^ Parents often report that their child’s condition seems to decline rapidly, but it is important to discuss symptoms or behaviours exhibited in the day(s) prior to previous exacerbations. On reflection, parents may be able to identify non-specific signs (such as decreased dietary intake, runny nose) as a precursor to an attack. Recognition of the signs of increased work of breathing should be discussed with parents and thresholds set for medical review. This may need to be adapted dependant on family dynamics, geographical location and severity of previous attacks. Safety is paramount and parents should not be made to feel they are over reacting or seeking too many medical reviews; they must feel confident to seek help at crucial times.

## Creating a healthy environment

This is a 3-year old child with recurrent episodes of wheezing. The health care professional has a responsibility to help parents create a healthy environment by addressing any modifiable risk factors such as tobacco and biomass smoke, in-door allergens, house dampness and also to provide information about inevitable respiratory viral triggers of asthma exacerbations.

### Environmental risk factors for asthma exacerbations

The link between the environment and exacerbation of asthma symptoms is a well-described entity.^8^ Many studies have described the role of air pollutants (indoor and outdoor) including biomass smoke and fumes from cars and factories, in triggering asthma symptoms.^36, 37^ The effect of environmental tobacco smoke, also known as second-hand smoking in causation and exacerbation of asthma symptoms in children is also well-documented.^38–40^

Children exposed to environmental tobacco smoke, experience more frequent and severe exacerbations of the asthma symptoms, even where medical treatment is adequate.^37, 40^ The dust and surfaces in a smoker’s home have been found to be contaminated with tobacco smoke, even when parents avoid smoking in the house.^41^ Vapour phase nicotine and particulates have been also found in the home of smokers.^42, 43^ Generally, contamination and exposure to second-hand smoke are 5–7 times higher in the homes of ‘smoking outdoor’ people compared to non-smokers.^41^

Indoor air pollution, including use of biomass smoke from burning wood, animal dung and crop residues for cooking and heating, has been associated with an increased risk of asthma exacerbations in children and adults.^36^ Therefore, improving air quality at home and reviewing some of the activities that may trigger attacks will be an important aspect of creating a health environment for this child.

Besides air pollution, a consistent association of dampness with respiratory symptoms is found among both atopic and non-atopic children. House dust mite exposure and sensitisation may contribute, but the link seems to be related principally to non-atopic mechanisms.^44^ Moreover, indoor allergens from mouse, cats, pets, dust mite and mould have been described as important exposures that lead to exacerbation of asthma symptoms.^44, 45^

### What can be done to create a healthy environment for this child?

As the diagnosis of asthma is increasingly likely, ICS along with addressing modifiable environmental risk factors for exacerbations (particularly tobacco smoke), can reduce hospital visits, avoid high healthcare costs and improve quality of life of the child and his parents.^46, 47^ The parents need to understand the benefits of the lifestyle changes and should be motivated to creating a smoke-free home, without exceptions for guests or friends.

Smoking cessation is a complex process and the parents will need support from family, friends and the healthcare system to be able to stop smoking. Key to this process is an understanding the barriers to smoking cessation such as; parental beliefs about second-hand smoke and readiness to quit smoking. Stress has been described as a major barrier to quitting because cigarette smoking is often used to give (temporary) relief from stress.^48^ It is therefore important to discuss the sources of stress and coping strategies that are not harmful. It is also important to build on known motivators for smoking cessation including family support and the will to protect the child from the effects of tobacco smoke.^49^

Reduction in exposure to biomass smoke can be achieved through use of alternative cooking and heating fuel such as liquefied gas or by using improved cookstoves.^50^ However, the challenges in adopting such changes including costs involved and behavioural aspects must be discussed with the parents.^51^

Many children are sensitised to more than one allergen, and many households have damp rooms. Reducing exposure to damp and mould improves asthma control in adults, but the benefit of interventions such as regular cleaning, avoiding use of carpets, and withdrawing pets from the home,^43^ is described as ‘limited’ in guidelines and can be ‘expensive and complicated’.^8^

The story continues…

- The family doctor recognised that the parents needed time to discuss their concerns and to have answers to their questions. She spent the consultation listening to the story of admissions and on-going symptoms, and explained why there was uncertainty about the diagnosis and why the treatments that had been tried had not relieved all the symptoms. She arranged for the parents to meet with a specialist nurse who had expertise in managing pre-school children with asthma. At the review, the nurse was able to reinforce the information provided by the doctor, review (self) management strategies, offer practical advice on delivery of inhaled therapy, and discuss reducing environmental triggers (including offering the father support with smoking cessation). At a follow-up appointment a month later, although their son continued to have occasional symptoms he was still thriving, and the parents decided against another trial of ICS at this time. The parents felt reassured and supported, and the decision about a referral to a hospital clinic was postponed
